# A mixed-methods study investigating the potential and challenges of generic substitution of controlled substances in community pharmacies

**DOI:** 10.1016/j.rcsop.2025.100622

**Published:** 2025-06-06

**Authors:** I.M. Keller, J.M. Alexa, M.W. Meier, S.S. Allemann

**Affiliations:** a Pharmaceutical Care Research Group, Department of Pharmaceutical Sciences, University of Basel, Spitalstrasse 41, 4056 Basel, Switzerland; b Quality Management Consultant, Blisterman Consulting GmbH, Spiez, Allmendweg 21, 3705 Faulensee, Switzerland

**Keywords:** Controlled substances, Generic substitution, Bioequivalence, Community pharmacy, Pharmacy practice, Pharmacy research

## Abstract

**Background:**

Using generics became an established practice. Studies about dispensing practices of generic controlled substances are scarce.

**Objective:**

We investigated dispensing practices of generic controlled substances compared to non-controlled substances, challenges community pharmacists face when substituting them, and how they can be better supported.

**Methods:**

A mixed-methods approach was employed. We descriptively analyzed prescribing and dispensing rates of originals and generics of controlled and non-controlled substances. Ten community pharmacists were interviewed to investigate challenges and support options.

**Results:**

Seven hundred eight prescriptions were included in the data analysis. Physicians prescribed 54 % (167/307) of originals for controlled substances and 50 % (202/401) of originals for non-controlled substances (*p* > 0.05). A total of 37 % (62/167) of prescriptions for original controlled substances were substituted with generics in community pharmacies in contrast to 74 % (149/202) of prescriptions for original non-controlled substances (*p* < 0.001). Challenges mentioned by the interviewees included gaining trust in the context of generic controlled substance use, meeting patients' needs, and legal regulations. They named support measures, such as patient education by physicians, and reduction of the administrative workload.

**Conclusion:**

The analysis revealed a significantly lower substitution rate for controlled substances compared to non-controlled substances in pharmacies. Yet, physicians showed similar prescribing rates of originals and generics. The findings support the need to strengthen the collaboration between healthcare providers, and to improve education as well as awareness to ensure adequate patient care when substituting generic controlled substances.

## Highlights

• Mixed-methods approach enabled a comprehensive multi-faceted insight.
• Lower substitution rate for controlled substances compared to other medicines.
• Most frequently named challenge by pharmacists was the high administrative workload.
• More patient education by physicians is needed about generic controlled substances.

## 1 Introduction

Community pharmacies function as the main suppliers for prescription-only medicines and nonprescription medicines.^1^ In the daily work of community pharmacies, generic medicines play an increasingly important role.^2^ Generic medicines are bioequivalent counterparts to original medicines.^3^ The market authorization process requires the provision of data that demonstrates a comparable efficacy and safety. Replacing an original medicine with a generic equivalent is described as generic substitution.^4^ In many countries, pharmacists are allowed to replace a prescribed original medicine with a therapeutically equivalent generic version, but its implementation and legal frameworks vary.^5^ Generic substitution aims to foster the use of more cost-effective medicines and ensures a wider availability of bioequivalent medicines without compromising treatment effectiveness. Generic medicines can, therefore, contribute to the ease of a healthcare systemʾs overall financial burden and help bridge medicine supply shortages.^6^^,^^7^ Heiskanen et al., for example, observed that while 79.8 % of Finnish community pharmacies that were included in their sample faced almost daily supply shortages, these shortages rarely resulted in issues since enough alternative medicines were available for substitution.^8^ In England for instance, community pharmacists have limited authority to substitute medicines prescribed by physicians. However, under specific circumstances such as medicine shortages, substitution is permitted, which is regulated through the Serious Shortage Protocols (SSPs).^9^

Previous research has suggested that generic substitution of medicines for the nervous system may be especially difficult. A literature review by Blier et al., for instance, has shown that switching between original and generic medicines may lead to severe consequences for patients with psychiatric disorders.^10^ Lang et al. found that switching medicines from different manufacturers increased the risk of seizures in patients, based on a retrospective data analysis.^11^ The challenges observed in the substitution of antiseizure medicines or psychiatric medicines show how difficult the therapy of neurological disorders is. Controlled substances are also used to treat seizures, anxiety, attention deficit hyperactivity disorder (ADHD), and pain.^12^ This demonstrates the importance of understanding how generic substitution for controlled substances is managed and the challenges it may present.

Research conducted by El-Haïk et al. in France established a positive attitude towards the generic substitution of controlled substances. In a qualitative study, healthcare professionals agreed that generic substitution is an important factor for reducing healthcare costs, as long as generics provide an equivalent alternative to the original medicine. However, they mentioned that careful consideration is required when treating patients with opioid use disorders. Additionally, healthcare professionals indicated that the substitution of buprenorphine is particularly beneficial for naive or stable patients.^13^ In contrast, institutions in certain countries recommend against the substitution of controlled substances. Gozzo et al. conducted a critical analysis for generic substitution in Italy, including medicines with a narrow therapeutic index. Based on their findings, they concluded that patients who undergo long-term opioid therapy may experience reduced effectiveness when switching to generics. Due to these concerns, some centrally acting opioid analgesics are now classified as non-interchangeable in Italy.^14^

Furthermore, modified-release formulations also play an important role in the substitution process. Seoane-Vazquez et al. analyzed the interchangeability of modified-release formulations of controlled substances and highlighted the need for caution when switching manufacturers. Extended-release formulations pose additional challenges for establishing bioequivalence compared to immediate-release drugs.^15^ A randomized, double-blinded, cross-over, single-center study conducted by Fallu et al. demonstrated patient satisfaction with the original compared to the generic formulation of a long-acting methylphenidate for example.^16^

In Switzerland, the Swiss Agency for Therapeutic Medicines (Swissmedic) follows established guidelines that require generics to have the same qualitative and quantitative composition of active substances as the original medicine.^3^^,^^17^ Switzerland bases its Swiss Narcotics Act (NarcA)^18^ on the International Narcotic Control Board (INCB). The INCB provides guidelines to help countries manage controlled substances for medical and scientific use while preventing misuse. The Single Convention on Narcotic Drugs classifies substances into different directories based on their risk of abuse and therapeutic value. Control measures vary by directories, with stricter controls on higher-risk substances.^19^^,^^20^ In Switzerland, opioid analgesics and psychostimulants are classified in list a, while other substances, such as benzodiazepines, are classified in list b.^18^^,^^21^ All controlled substances classified in Switzerland under list a and b comprise medicines for the nervous system.^22^

Previous studies on the generic substitution of controlled substances have not dealt with prescribing practice of physicians or the dispensing practices of community pharmacists. This lack of data hinders comparisons with prescribing and dispensing practices in non-controlled substance classes. Such comparisons would provide additional understanding of the challenges faced in daily pharmacy practice, including those posed by regulatory barriers, uncertainties among community pharmacists, and patients' habits as well as concerns. Therefore, this study aimed to explore the use of generic controlled substance medicines in Switzerland. The two aims of this study were: 1. To investigate dispensing practices of generic controlled substances in community pharmacies as well as physicianʾs prescribing practice based on a retrospective prescription analysis, and 2. to identify community pharmacistʾs challenges and support needed when substituting generic controlled substances.

## 2 Methods

### 2.1 Study characteristics

#### 2.1.1 Study design

A mixed-methods approach was employed. First, a quantitative prescription analysis was used to determine the substitution rate of generic controlled substances in comparison to other medicine classes that are not considered as controlled substances in Switzerland. Second, qualitative interviews were conducted to gain an insight into the challenges community pharmacists face when substituting generic controlled substances and potential strategies to overcome these.

#### 2.1.2 Recruitment of the study population

Nine different community pharmacies were invited by e-mail between February 24th, and March 6th, 2023. Participating pharmacy managers were asked to provide their informed consent. The prescription analysis and the interviews were executed between March 3rd, and March 24th, 2023.

#### 2.1.3 Study area

Community pharmacy managers from community pharmacies located in the German-speaking part of Switzerland were invited to participate. Switzerland is divided into four distinct language regions: German-speaking, French-speaking, Italian-speaking, and Romansh-speaking Switzerland. Community pharmacies from a diverse range of locations (rural and urban areas) were chosen to cover a variation of prescriptions received for analysis and interview partners.

#### 2.1.4 Ethics approval

This study did not fulfill the criteria for research according to the Swiss Human Research Act and therefore was exempt from ethics approval from a competent cantonal ethics committee. The study was approved following internal review at the Department of Pharmaceutical Sciences of the University of Basel. It was ensured that all data collected was fully anonymized and handled in compliance with Swiss data protection regulations, thereby adhering to ethical research standards.

### 2.2 Quantitative data analysis

#### 2.2.1 Prescription analysis

**Fig. 1:**
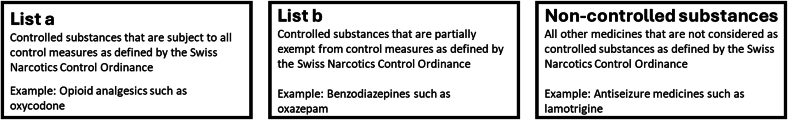
Classification of controlled substances into list a and list b and non-controlled substances in Switzerland according to the NarcA and Swiss Narcotics Control Ordinance (NarcCO).^18^^,^^21^

Each community pharmacy was asked to provide 20–25 randomly chosen prescriptions for controlled substances that fall under list a and b as well as 50 randomly chosen prescriptions of non-controlled substances (Fig. 1). The information was extracted in a coded format and limited to the prescribed product (original, generic, or active substance) and the dispensed product (original or generic), ensuring no patient or physician data were revealed.

#### 2.2.2 Statistical analysis

We analyzed the substitution rate by community pharmacists and the prescribing rates by physicians as dependent variables, while the substance class (controlled and non-controlled) served as the independent variable. The analyzed data included variables with expected frequencies above 5. A Pearson Chi-Square test was performed to determine whether the substitution rates of community pharmacists differ significantly between generic controlled substances (list a and list b) and non-controlled substances. Additionally, the generic prescribing rates of physicians for these medicine classifications were assessed. The threshold for statistical significance was set at *p* ≤ 0.05. The Phi coefficient was used to assess the association between the variables studied (list a, list b, and non-controlled substances). According to Cohen, a value of 0.1 represents a small effect, 0.3 a medium effect, and 0.5 a large effect.^23^ SPSS® (IBM SPSS Statistics, version 29.0.0.0 (241)) and Microsoft Excel® (Microsoft 365, 2016) were used for the data analysis. To ensure transparency, we present the findings of our quantitative analyses in accordance with the Strengthening the Reporting of Observational Studies in Epidemiology (STROBE) guidelines.^24^

### 2.3 Qualitative data analysis

#### 2.3.1 Expert interview

A semi-structured questionnaire was used to navigate the interview process, while allowing flexibility when needed. The questionnaire aimed to identify the challenges community pharmacists face in substituting controlled substances and the support they need to manage this process. The questionnaire included five open-ended questions that addressed generic substitution, as well as two closed-ended questions concerning sociodemographic data. The interviews were conducted in Standard German. Interviewees were not informed about the topic of the questionnaire in order to explore whether community pharmacists viewed generic substitution of controlled substances as challenging. Time until completion was estimated to account to up to 60 min.

The first question functioned as an icebreaker question to explore the interviewee's attitude towards generic substitution. The second question addressed which medicines are viewed to be difficult to substitute with a generic medicine. Further questions intended to reveal what challenges community pharmacists face in everyday practice and what support is desired to enhance the uptake of the substitution of generic controlled substances. The translated interview questions can be found within the Appendix A.1.

#### 2.3.2 Interview analyses

Data was collected by one interviewer (IMK) during face-to-face meetings in a separate room at each participating community pharmacy. The interviews were recorded as audio files, with an audio recording device provided by the University. The audio recordings were transcribed verbatim. Once transcribed, the recordings were deleted. The qualitative data were analyzed using an inductive approach according to Kuckartz's structured qualitative content analysis.^25^ Overarching categories and subcategories were formed according to Kuckartz's structured qualitative content analysis. The data were analyzed using the MAXQDA 2024 (VERBI GmbH, Berlin, Germany, version 24.3.0). To ensure proper intercoder reliability, two researchers (IMK, JMA) initially coded one interview and created a coding tree. The remaining interviews were then coded individually by one of the authors based on the coding tree and then reviewed by the other author. All changes were documented in the MAXQDA LogBook. Questions or uncertainties were discussed and adjusted afterwards. The quotes used in this publication were translated from German to English using DeepL Translator (DeepL SE, Köln, Germany, Version 24.8.1.). To ensure transparency in the reporting of qualitative data, the Standards for Reporting Qualitative Research (SRQR) list and the Consolidated Criteria for Reporting Qualitative Research (COREQ) 32-item checklist were used as guidance.^26^^,^^27^

## 3 Results

All nine contacted pharmacies agreed to participate and provide prescriptions for the prescription analysis. Ten pharmacists, with a median age of 53.6 years, agreed to participate and gave informed consent for the interviews. Two interviewees shared the management of one community pharmacy and were, therefore, interviewed simultaneously. Details about the characteristics of all participating community pharmacies and interviewees are shown in Table 1.

**Table 1:** Demographic data of the community pharmacies and interview participants.

Demographic Data of the Community Pharmacy	Number of pharmacies (*n* = 9)
Location characteristics: • rural • suburban areas • urban	44.44 % (4) 11.11 % (1) 44.44 % (4)
Pharmacy location: • inside a mall • next to a shopping center • pedestrian zone • neighborhood/quarter	33.33 % (3) 22.22 % (2) 11.11 % (1) 33.33 % (3)
Pharmacy structure: • chain • privately owned within a franchise network	55.56 % (5) 44.44 % (4)

Demographic Data of the Interview Participants	**Number of participants** **(*n*** **= 10)**

Gender: • female • male	30 % (3) 70 % (7)
Highest academic degree: • Doctorate • Swiss additional License for practicing as a specialist in community pharmacy • Swiss Federal Pharmacy Diploma (Pharmacy Practicing License)	30 % (3) 50 % (5) 20 % (2)
Graduation time intervals (Reception of Swiss Federal Pharmacy Diploma): • < 1990 • 1990–2000 • 2001–2010 • >2010	30 % (3) 30 % (3) 20 % (2) 20 % (2)

### 3.1 Quantitative analysis of prescription data

**Fig. 2:**
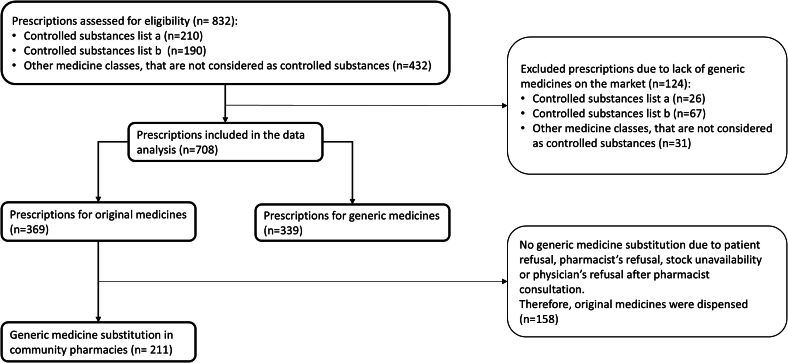
Prescription analyses flowchart. Display of excluded and included prescriptions, number of generic and original prescriptions, generic substitution and original dispense.

Prescription data from nine community pharmacies were analyzed after giving informed consent. A total of 832 prescriptions with prescribed active substances that are covered by the obligatory Swiss health insurances were assessed for eligibility. After excluding prescriptions due to lack of generics on the market, we included 708 prescriptions for analysis (Fig. 2).

Each community pharmacy provided 35 to 50 prescriptions for controlled substances, including 20 to 25 prescriptions of substances, which are subject to all control measures (list a) and 10 to 25 prescriptions of list b controlled substances. Additionally, 50 to 62 prescriptions for non-controlled substances per pharmacy were analyzed.

### 3.2 Generic prescription rate by physicians

**Fig. 3:**
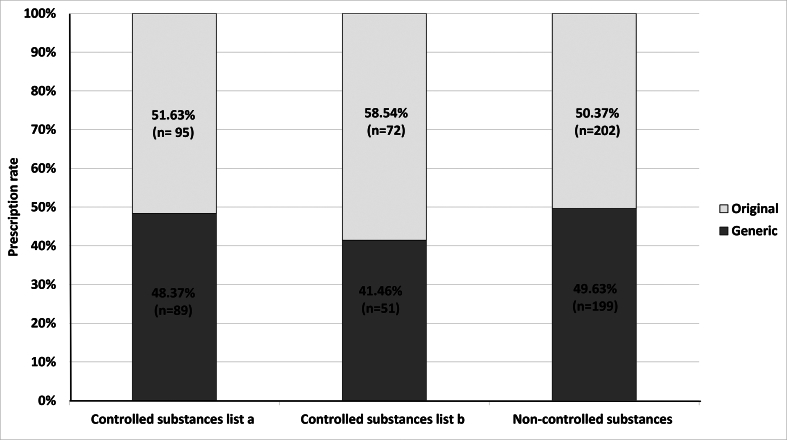
Absolute prescribing rates and relative prescribing rates of generic and original prescriptions issued by physicians. These prescriptions were filled in community pharmacies.

Out of 708 prescriptions included in the data analysis, 307 prescriptions for controlled substances contained 167 (54 %) prescribed original medicines. The remaining 401 analyzed prescriptions of non-controlled substances on the other hand consisted of 202 (50 %) prescribed original medicines. Of the prescriptions for controlled substances, 89 (48 %) and 51 (41 %) were for substances from list a and b, respectively (Fig. 3). No statistically significant differences in physicians' prescribing of generic versus original medicines were detected over the three compared classes (Table 2).

**Table 2:** *P*-values and Phi coefficient, calculated using the Pearson Chi-Square test, showed differences in substitution rates between community pharmacies and physicians' generic prescriptions across the listed medicine classes. Asterisks (*) indicate statistically significant values (*p* ≤ 0.05).

Compared classes		Substitution rate in community pharmacies	Prescribed generics by physicians
Controlled substances list a and controlled substances list b	*p-value*	0.831	0.234
	*Phi coefficient*	0.018	−0.068
Controlled substances list a and non-controlled substances	*p-value*	< 0.001*	0.778
	*Phi coefficient*	−0.345	0.012
Controlled substances list b and non-controlled substances	*p-value*	< 0.001*	0.113
	*Phi coefficient*	−0.345	0.069

### 3.3 Generic substitution rate of prescriptions in community pharmacies

**Fig. 4:**
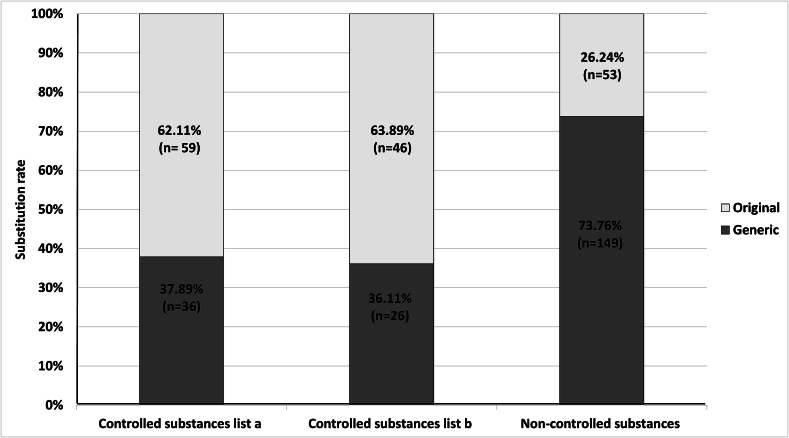
Absolute substitution rates and relative substitution rates of generics in relation to all issued original prescriptions in community pharmacies.

Community pharmacists substituted 62 (37 %) generics out of 167 analyzed prescribed original medicines for controlled substances and 149 (74 %) generic medicines out of 202 original prescriptions for non-controlled substances (Fig. 4). The substitution rates for prescriptions of non-controlled substances differed significantly from both controlled substance classifications (list a and list b). The Phi coefficient indicated in both comparisons (list a vs. non-controlled substances and list b vs. non-controlled substances) a medium strength of association between the classes (*p* ≤ 0.001 and *Phi =* −0.345). No statistically significant difference in generic substitution rates between controlled substances list a and controlled substances list b was detected (*p* = 0.831). Additionally, the strength of association between list a and list b was weak (*Phi* = 0.018). All results regarding the comparison between substitution rates are displayed in Table 2.

#### 3.3.1 Qualitative analysis of interview data

Based on the qualitative data, three groups of content categories were formed: Medicine classes that are difficult to substitute, challenges when substituting controlled substances, and support for the generic substitution of controlled substances. Within these three groups, eight overarching content categories were identified, to which letters were assigned (A to H). These were further subdivided and numbered into specific content subcategories (1 to max. 10). The content subcategories were likewise further divided when required and assigned numbers (1 to 2). The quotes mentioned in the following text are referenced based on the category they belong to in the coding tree. (Example of a detailed reference; content group 2 / overarching content category B / specific content subcategory 3 / finer content sub-subcategory 1). The full coding tree can be found within the Appendix B.1.

#### 3.3.2 Medicine classes that are difficult to substitute

The interviewees named different medicine classes that are difficult to substitute. Only two community pharmacists spontaneously mentioned controlled substances as difficult to substitute. One interviewee explicitly mentioned methylphenidate as difficult to substitute because of the different kinetic properties of original and generic products. After multiple inquiries about their approach, one community pharmacist highlighted that a consultation with the prescribing physician is always undertaken when dealing with list a controlled substances. The interviewee said: *“In any case, because it is a controlled substance, when we want to change something on the prescription and do not exactly proceed as written on the prescription, then we want to hold a consultation because it is a delicate therapy […]”* (1/A/9).

Among all medicine classes, antiseizure medicines were most often mentioned as difficult to substitute. As one interviewee put it: *“Then there are some substances that are generally never substituted, such as antiepileptic medicines with a narrow therapeutic range, where small changes in bioavailability can have a major impact and thus affect therapy”* (1/A/2)*.* Multiple pharmacists reported that they consult with the prescribing physician when a generic substitution is considered in the therapy of epilepsy. Furthermore, six interviewees mentioned psychiatric medicines as being challenging for generic substitution. Moreover, it was mentioned that psychiatrists often refuse a generic substitution and that patients also often express concerns regarding changes in their therapy. This is illustrated by the following quote: *“Where it is difficult in general, even though one could do it, actually pharmaceutically (speaking) without problems, are psychopharmacological medicines, such as antidepressants first and foremost. But there is often the customer, who is psychologically distressed, he has too much fear to switch”* (1/A/8). Cytostatics were also mentioned as challenging to substitute by three community pharmacists. They stated that patients are usually well-adjusted to their current therapy and should not undergo a generic substitution under these conditions.

#### 3.3.3 Challenges when substituting controlled substances

The interviewees identified various challenges in the general process of generic substitution. Opinions differed concerning nursing homes. One interviewee mentioned that the generic substitution of nursing home patients is difficult. Changes in prescriptions may be challenging for different staff on duty. One interviewee, on the other hand, considered generic substitution in nursing homes to be easier compared to patients living at home. Additionally, three interviewees emphasized the importance of the patient's acceptance and adherence, which were viewed as more crucial than generic substitution. For example, one interviewee said: *“[…] I of course want the compliance to be optimal, especially for those who come in every day, we look after them closely and want to have the acceptance. We would do it after the consultation with the customer and the physician […]”* (2/B/2).

Furthermore, challenges related to the substitution of generic medicines in the treatment of nervous system disorders were identified. Five community pharmacists highlighted that patients who become accustomed to the original medicine pose a challenge. Patients have concerns about a potential loss of effectiveness in their pain management therapy, therapy with central stimulants, or sleeping aid therapy. One interviewee put it as follows: *“There are certainly patients who are adjusted and take it for a long time, a therapy with an original product, and want to stay on it. For various reasons, even if they are told that the generic is an equivalent medicine. They want it in that way, so that they are granted to be well adjusted, and it helps to ease the pain […]”* (2/C/1/1).

The interviewees' responses regarding the generic substitution of controlled substances varied. Community pharmacists stated that physicians usually write the name of the original controlled substance product on the prescription. Furthermore, their description of the cooperation with physicians regarding controlled substances varied. Five reported to have experienced difficulties, whereas one described communication with physicians as unproblematic. Additionally, six community pharmacists reported that bureaucracy is one of the major challenges in substituting controlled substances in general pharmacy practice. The following comment illustrates this: *“Basically, the prescriptions with controlled substances are more bureaucratic in every perspective and that may also be a reason why it is more difficult to substitute […]”* (2/D/2).

Two community pharmacists stated that pharmacy technicians may exhibit reluctance to address generic controlled substance substitution during a consultation, as exemplified by the following statement: *“Dealing with these strictly controlled substances is sometimes a bit difficult so that some people do not have enough confidence. I mean, we have pharmacy technicians and trainees; they are usually the people at the frontline, and if they don't have the confidence […]. The thought of a separate law for controlled substances and that they should, therefore, be handled more strictly […]”* (2/D/4). In contrast, four pharmacists mentioned that there were no challenges when substituting opioid analgesics. Meanwhile, four community pharmacists expressed no concerns regarding the loss of effectiveness when substituting controlled substances. Another four saw no scientific basis for avoiding such substitutions.

### 3.4 Support for the generic substitution of controlled substances

Participants suggested different ways, how they would like to be supported in the daily practice of substituting medicines. A standardized note within the prescription, such as “Generics preferred” by physicians, was suggested as a potential aid. Four interviewees also mentioned information about available co-marketing medicines as helpful. The availability of generic medicines and medicine stock management were also named as important factors for the implementation of generic substitution.

The community pharmacists did not mention support needed when substituting medicines used to treat nervous system disorders. However, two interviewees highlighted the importance of a list provided by a generic manufacturer. The list contains information on active substances used in the treatment of nervous system disorders and their substitution conditions. One interviewee said: *“We have lists, for example, from the manufacturer […], which indicate sensitive products, like antiepileptics, where caution is needed, especially when they are used to treat epilepsy”* (3/G/1).

Of interest is the demand for a list that would provide information on substitution conditions of controlled substances, which is not yet available. In total, six community pharmacists expressed the need for official information when substituting controlled substances. As stated by one interviewee: *“The question is, if we are allowed to write directly on the prescription for controlled substances or if a new one has to be sent? Yes, I would also need information on what I am officially allowed to do”* (3/H/3). Further, data regarding quality, safety, and equivalence of generic products for patients at community pharmacies were demanded. Two community pharmacists suggested that physicians should provide more information about substituting in the class of controlled substances when consulting patients. The reduction of the administrative burden was, furthermore, reported as a form of support that would ease generic substitution of controlled substances.

Furthermore, three interviewees mentioned that an increase in cooperation between healthcare providers based on the controlled substances would be helpful. The experience and time to become familiar with generic controlled substance medicines remains an important aspect.

## 4 Discussion

Very little was found in the literature about the generic substitution of controlled substances and the challenges community pharmacists face when substituting controlled substances. Therefore, this study seeks to address the generic medicine substitution practice and to identify community pharmacistsʾ challenges as well as support options. The prescription analysis revealed that while physicians prescribed nearly equal amounts of original and generic medicine in all compared medicine classes, community pharmacists substituted controlled substances significantly less frequently compared to non-controlled substances. Community pharmacists highlighted the need of physicians to inform their patients about generic substitution. This would provide support for community pharmacists in further consulting patients, especially when substituting controlled substances.

The substitution behavior of community pharmacists differed from the generic prescribing practice of physicians. Interestingly, the substitution rate of controlled substances (37 %) in comparison to non-controlled substances (74 %) was significantly lower in community pharmacies. However, a few interviewed community pharmacists described controlled substances as unproblematic for substitution from a pharmaceutical perspective; no concerns regarding a loss of effectiveness were mentioned. Nevertheless, the difference in substitution rates may be attributed to pharmacists' uncertainty regarding legal restrictions or specific requirements associated with substituting controlled substances. From a legal perspective, there are no restrictions that prohibit the generic substitution of controlled substances according to the Therapeutic Medicines Act (TheraMA) and the NarcA.^18^^,^^28^ Given an appropriate documentation, substitution of controlled substances is permitted. Challenging bureaucratic requirements, on the other hand, were named as one of the barriers to generic substitution by the interviewees. These time-consuming administrative efforts that pharmacists are confronted with in daily practice could further explain why the substitution rate of controlled substances was lower than the substitution of non-controlled substances.

The Medical Practice and Quality Committee of the American College of Physicians recommended to reduce administrative burden in the healthcare sector. Recommendations such as simplifying documentation requirements and developing suitable automation technologies are included.^29^ An ease of the bureaucratic burden may also simplify the processes in Switzerland and, therefore, be beneficial. Such an effort could also be implemented in the generic substitution process of controlled substances, as it would simplify the administrative burden in community pharmacies. The community pharmacists outlined further reasons for refusal of generic substitution by patients. They stated for instance that patients in difficult life situations are not interested about saving costs. They usually take what is prescribed or what is already in pharmacy stock to get the medicine as fast as possible. This suggests that the availability of generics in pharmacy stock may facilitate patient acceptance of generics. These findings regarding patient refusal are consistent with earlier observations by Nokelainen et al., who identified two primary reasons for patients' refusing generic substitution. The first was minor price differences between original and generic, and the second was that the previously used medicine was considered good.^30^

For prescribing physicians, the bureaucratic burden and patient refusal associated with prescribing generic controlled substances appears to be minimal. This may explain the surprisingly similar amount of prescribed original and generic medicines for both controlled substances (46 %) and non-controlled substances (50 %). This outcome contradicts the statement made by community pharmacists, who indicated that physicians usually write down the name of the original medicine on the prescription regarding controlled substances. However, community pharmacists reported challenges when communicating with physicians, who primarily prescribe original products of controlled substances. In the case of prescription-only medicines, a physician is typically the patient's initial point of contact. The community pharmacists highlighted that patients are often not adequately informed by physicians about the possibility of substituting controlled substance medicines with generics. This includes a written note stating “Generics preferred” on prescriptions in general. This initiative could be very helpful for community pharmacists and increase the uptake of generic substitution. The note would also provide reassurance to patients, especially when substituting controlled substances.

In addition to the information provided by physicians, the confidence of community pharmacists in generic medicines is important. Drozdowska and Hermanowski collected data through face-to-face interviews with patients in Poland and revealed that the opinions of pharmacists and physicians play an essential role in patients' decisions.^31^ This finding undermines the importance of official information during the consultations, which supports the generic substitution of controlled substances in community pharmacies. These results are in alignment with previous research, such as by Xanthopoulou and Katsaliaki, who analyzed the patients' and physicians' opinions about generic dispensing in Greece. Physicians' trust in generic medicine manufacturers was the decisive factor in prescribing generic medicines. The authors recommended promoting communication between physicians and the pharmaceutical industry to earn patients' trust.^32^ Increased efforts by generic manufacturers are necessary to improve the dispensing of generic controlled substances, even though some community pharmacists reported no difficulties in substituting controlled substances. The quantitative data shows significantly lower substitution rates for controlled substances compared to non-controlled substances by community pharmacies. This unexpected contrast underlines the importance of effective collaboration between the pharmaceutical industry, physicians, and community pharmacists.

Studies by Schackmann et al., and Haskard Zolnierek and DiMatteo have shown that a healthcare professional's well-practiced conversation and empathetic, honest, and confident appearance can positively influence patients' decisions. Strong communication and clarity in the consultation provided by healthcare professionals can facilitate a switch to generics and promote adherence. Patients at the same time need the opportunity to express emotions and fears during consultations. The primary goal of a healthcare professional during a consultation should be to seek a solution collaboratively with the patient.^33^^,^^34^ This states the importance of community pharmacists' and pharmacy technicians' competent and confident communication. The consideration of psychological aspects and patients' habits remains essential, and it outlines the necessity of a professional, informative, and trustworthy consultation by community pharmacists and pharmacy technicians.

Further challenges, especially in substituting medicines for neurological disorders, were highlighted by the interviewees. Nearly all interviewees reported antiseizure medicines as particularly risky and unlikely to be substituted, especially without prior consultation with a physician. As outlined by Maliepaard et al., the regulatory requirements for generic medicines ensure bioequivalence and identical absorption of the active substance between the generic and the original medicine. This guarantees equivalent therapeutic effectiveness in patients. The effect is proven based on the equivalent absorption, leading to an equivalent circulation of the active substance and hence no plausible difference between a generic and an original. Variations in individual patient plasma are possible but similarly affect both generic and original medicines.^35^ As participating community pharmacists mentioned, patient adherence is crucial and ultimately probably more important than the aspect of substitution itself. As analyzed by Kesselheim et al., variations in pill appearance can exacerbate non-adherence and adversely affect outcomes in evaluations of generic medicines. In conclusion, the variety in colors and shapes can increase patients' insecurity and the risk of interrupting medicine use.^36^ This leads to the observation that a similar appearance of the pills could facilitate patient adaptation to a change. Additionally, interviewees highlighted patient-related obstacles when substituting medicine for neurological disorders, including patients' habits and concerns about the effectiveness of generics.

### 4.1 Strengths and limitations

The main strength of the current study lies in its use of a mixed-methods approach. This methodology enabled a comprehensive and multi-faceted insight. By analyzing quantitative data, the results were strengthened, and the influence of social desirability bias among the interviewees was minimized. The quantitative phase of the study allowed gaining a deeper understanding of the status quo of dispensing generic medicines in defined medicine classes. The qualitative phase of the study helped to better understand what challenges may play a role when substituting controlled substances. Furthermore, the study describes potential support strategies in daily pharmacy practice that could help to improve the generic substitution of controlled substances in the future.

However, this study has a few limitations. The data are limited to nine community pharmacies in the German-speaking region of Switzerland. The results may not accurately reflect the practices of all pharmacies in Switzerland and worldwide. Analyses in all regions would have been useful for a more comprehensive understanding. Such an approach would allow the exploration of regional similarities and differences regarding the challenges and support mentioned. Second, the study did not investigate the underlying reasons for the dispense of original medicines instead of generic medicines. Third, the interview data analysis revealed that substituting medicines for nervous system disorders is particularly challenging. An opportunity for further research could involve analyzing the prescription and substitution rates of these medicine classes, which would allow a comparison with controlled substances. Additionally, a cross-national mixed-methods study involving physicians, community pharmacists, and patients is needed to identify further challenges, determine required support, and ensure adequate patient care.

## 5 Conclusion

This study highlights the potential for improvement in the substitution of controlled substances and reveals several important clinical implications. The current findings support the relevance of collaboration between pharmacists, physicians, regulatory authorities, pharmaceutical companies, and patients. Overall, this study revealed that there is a need to develop effective strategies that are tailored according to pharmacistsʾ needs to optimize patient care and improve substitution practice of controlled substances. Further research on the generic substitution of controlled substances would provide a deeper insight into the topic.

## CRediT authorship contribution statement

**I.M. Keller:** Writing – original draft, Visualization, Project administration, Methodology, Investigation, Formal analysis, Data curation, Conceptualization. **J.M. Alexa:** Writing – review & editing, Formal analysis. **M.W. Meier:** Writing – review & editing, Supervision, Conceptualization. **S.S. Allemann:** Writing – review & editing, Supervision, Resources, Conceptualization.

## Declaration of generative AI and AI-assisted technologies in the writing process

This publication used DeepL Translator (DeepL SE, Köln, Germany, Version 24.8.1) to translate specific text passages from German to English. Additionally, ChatGPT (OpenAI, San Francisco, CA, USA, Version 3.5) and Grammarly Premium (Grammarly Inc., San Francisco, CA, USA, Version 1.2.124.1571) were used for language editing, enhancing style consistency, clarity, and readability. After using these tools, the authors reviewed and edited the content as needed and take full responsibility for the content of the published article.

## Declaration of competing interest

Ioana Maria Keller reports financial support was provided by Medifilm AG. Markus Werner Meier reports financial support was provided by Medifilm AG. If there are other authors, they declare that they have no known competing financial interests or personal relationships that could have appeared to influence the work reported in this paper.

Markus W. Meier and Ioana M. Keller are employed by Medifilm AG, which provided salary support during the research period. The company had no involvement in the study design, data collection, analysis, or interpretation, and the study was not sponsored by Medifilm AG. All authors declare no financial or non-financial competing interests.

## Data Availability

The data that support the findings of this study are available from the corresponding author upon reasonable request.

## Appendix A Supplementary data

Supplementary material 1 Image 1

Supplementary material 2 Image 2
