# Supplementary material for: A mixed-methods study investigating the potential and challenges of generic substitution of controlled substances in community pharmacies
Source: Explor Res Clin Soc Pharm. 2025 Jun 6;19:100622. doi: 10.1016/j.rcsop.2025.100622 (PMC12206124; doi:10.1016/j.rcsop.2025.100622)
Supplement: Supplementary file 1 — Supplementary material 1 [file mmc1.pdf]

Semi-structured expert interview questionnaire for community pharmacists

<table>
<tr><th>Leading question</th></tr>
<tr><td>1. What experiences have you had in everyday pharmacy practice regarding generic substitution?</td></tr>
<tr><th>Specific follow-up questions</th></tr>
<tr><td>• What experiences have you had with physicians when substituting at the community pharmacy?
• Which prescription form (generic/original/active substance) is mostly chosen by the physicians?
• What experiences have you had with patients when you proposed a generic substitution at the pharmacy?</td></tr>
</table>

<table>
<tr><th colspan="2">Leading question</th></tr>
<tr><td colspan="2">2. Tell me which medicine class you would avoid substituting in everyday practice?</td></tr>
<tr><th>Maintaining the question</th><th>Specific follow-up questions</th></tr>
<tr><td>• Which additional medicine class would you not substitute?
• Which medicine class do you consider challenging to substitute?
• If we focus on the Anatomical Therapeutic Chemical (ATC) Classification of the Nervous system, which medicine class do you consider challenging to substitute?</td><td>• How do you perceive the generic substitution of controlled substances?
• What experiences have you had with the substitution of controlled substances?</td></tr>
</table>

<table>
<tr><th colspan="2">Leading question</th></tr>
<tr><td colspan="2">3. Based on internal data, we can observe that in 2022, we achieved a substitution rate of up to 74.3% defined daily dosage (DDD) in the generic market, while in the list a of the controlled substances, we are at 30.1% DDD how do you explain such a difference?</td></tr>
<tr><th>Maintaining the question</th><th>Specific follow-up question</th></tr>
<tr><td>• How do you explain that we have a substitution rate of 76.65% DDD list b of controlled substances?
• Which prescription form (generic/original/active substance) is increasingly being chosen?</td><td>• What differences do you see in the substitution of controlled substances list a compared to other medicine classes?</td></tr>
</table>

<table>
<tr><th colspan="2">Leading question</th></tr>
<tr><td colspan="2">4. What would you specifically need in order to substitute controlled substances list a more frequently?</td></tr>
<tr><th>Maintaining the question</th><th>Specific follow-up question</th></tr>
<tr><td>• What do you think your community pharmacy colleagues would need in order to substitute controlled substances more often?</td><td>• What support measures can you suggest that would be helpful in everyday practice to promote generic substitution of controlled substances?</td></tr>
</table>

<table>
<tr><th>Closed-ended Question</th></tr>
<tr><td>5. How long have you been practicing as a community pharmacist?</td></tr>
</table>

<table>
<tr><th>Closed-ended Question</th></tr>
<tr><td>6. How many controlled substance prescriptions list a do you have per week? (including refills)</td></tr>
</table>

Specific follow-up question
• How many controlled substance prescriptions list b do you have per week? (including refills)

Leading question
**7.** We have discussed a lot about controlled substances in everyday pharmacy practice. Is there anything from your side that has not been mentioned in the interview so far, but you consider important in this context?
**Specific follow-up questions**
• What other aspects come to your mind regarding controlled substances list a?<br>• What other aspects come to your mind regarding generic substitution?
