# Supplementary material for: A mixed-methods study investigating the potential and challenges of generic substitution of controlled substances in community pharmacies
Source: Explor Res Clin Soc Pharm. 2025 Jun 6;19:100622. doi: 10.1016/j.rcsop.2025.100622 (PMC12206124; doi:10.1016/j.rcsop.2025.100622)
Supplement: Supplementary file 2 — Supplementary material 2 [file mmc2.pdf]

**Content group 1**<br>**Main Question Topic:**<br>Medicines classes that are difficult to substitute (original vs. generic)				
A	**Overarching content category:**<br>Dispensing a generic instead of the original was described as difficult for:			
A	**Nr.**		**Specific content subcategories**	**Example-Quotes**
	1		Antiparkinson medicines	"[…] we should consult the physician, also Antiparkinson medicines could be problematic, depending on where it may be sensitive […]."
A	2		Seizure suppressants	"Then there are some substances that are generally never substituted, such as antiepileptic medicines with a narrow therapeutic range, where small changes in bioavailability can have a major impact and thus affect therapy."
A	3		Antihypertensives	"Also blood pressure-lowering medicines, when we are substituting and the customer agrees, I offer a free blood pressure measurement a few days after the changeover. […] We explain to them that the changeover to the generic medicine may cause a fluctuation until the changeover is stable."
A	4		Hormones	"[…] also hormones one knows, if there is a generic on the market, do not have the same effect, so we always consult the physician."
A	5		Antidiabetics	"[…] Diabetics, these people are often well adjusted in their therapy and then I call the physician to ask what he thinks about a substitution."
A	6		Cytostatics	"If it is a cytostatic medicine, where the substitution is really not simple […]."
A	7		Immunsuppressants	"[…] there are some framework conditions, especially for immunsuppressants […] there, substitution is only possible after consulting the respective physician."
A	8		Psychopharmacologic medicines	"Where it is difficult in general, even though one could do it, actually pharmaceutically (speaking) without problems, are psychopharmacological medicines, such as antidepressants first and foremost. But there is often the customer, who is psychologically distressed, he has too much fear to switch."
A	9		Controlled substances	"In any case, because it is a controlled substance, when we want to change something on the prescription and do not exactly proceed as written on the prescription, then we want to hold a consultation because it is a delicate therapy […]."
A	10		Analgesics	"Of course, one has to take a look at certain analgesics, the galenic form (of a medicine) from one manufacturer may act faster, another one slower; there are even differences between humans."
**Content group 2:**<br>**Main Question Topic:**<br>Challenges when substituting controlled substances				
B	**Overarching content category**<br>Challenges that generally arise when substituting with generic medicine			
B	**Nr.**		**Specific content subcategories:**<br>Concerning community pharmacists' responses	**Example-Quotes**
B	1		Sufficient persuasiveness to be able to dispense the generic instead of the original medicine	"I do not know, it must have something to do with the patient's pain and the fact that one does not want to do it differently […] What applies in general is that the patient wants something that is just as good and that works in other areas of indications. So, it only has to do with our power of persuasion. That we take a positive approach to this substitution."
B	2		Acceptance and adherence of patients is important	"[...] I of course want the compliance to be optimal, especially for those who come in every day, we look after them closely and of course want to have the acceptance. We would do it after the consultation with the customer and the physician. [...]."
B	3		Patient satisfaction at risk	"When it does not work as desired due to perhaps psychological reasons, then we have a very dissatisfied customer and we all want to avoid that. I think that is the worst thing because it is difficult to win back the customers' trust if you have tried to switch them to a generic and they are not satisfied, and it will be difficult to regain their trust. [...]."
B	4	**Nr.**	**Content sub-subcategories:**<br>Patients as an important factor in the decision-making process	**Example-Quotes**
B	4	1	Difficulties in nursing homes	"In these institutions, it is probably the case that one is more likely to stick with the original in order to increase patient safety, because there are several interfaces, the prescription in the office and the nursing staff working in nursing homes. These interfaces represent a hazard point, in order to create safety, it is more common to stick with what is actually prescribed. This could play a role, because of course a very high rate of inpatients have to obtain controlled substances, unfortunately."
B	4	2	Needs in difficult life situations	"[...] the patient is not here himself or he is in a desperate situation, i.e. terminal cancer, where only palliative care is provided, so the patient and his surroundings are in a very tense mental state and do not wish to discuss anything and the price does not matter [...]."
B	5	**Nr.**	**Content sub-subcategories:**<br>Stock management	**Example-Quotes**

B	5	1	Original in stock	"It may also have something to do with the fact that we did not have many methylphenidate prescriptions here, more are emerging and since we tend to have the originals in stock, we are also increasingly dispensing what is on the prescription because it is already there and the patient would also prefer the original. [...]."
B	5	2	Delivery problems/medicine shortages in general	"However, there was always the problem of availability, and in many cases substitutions had to be made. [...]."
C	**Overarching content category**<br>Challenges that arise when substituting medicine for the nervous system			
C	**Nr.**	**Specific content subcategories:**<br>Concerning community pharmacists' responses		
C	1	**Nr.**	**Content sub-subcategories:**<br>Patients as an important factor in the decision-making process	**Example-Quotes**
C	1	1	Getting used to the original medicine	"There are certainly patients who are adjusted and take it for a long time, a therapy with an original product, and want to stay on it. For various reasons, even if they are told that the generic is an equivalent medicine. They want it in that way, so that they are granted to be well adjusted, and it helps to ease the pain. […]."
C	1	2	Fear of decreased effectiveness and trust in the original medicine	"In the case of methylphenidate, we also operate in an emotional environment. Parents, in particular, are skeptical if they fear that the success of the therapy could have an impact on the child´s performance at school or otherwise. […]."
D	**Overarching content category**<br>Challenges that additionally arise when substituting controlled substances			
D	**Nr.**	**Specific content subcategories:**<br>Concerning community pharmacists' responses		**Example-Quotes**
	1		Fear of decreased effectiveness of generic opioid analgesics	"Now, many colleagues are perhaps very reticent. [...] Because of this retention, because one thinks controlled substances are tricky and then, yes, he always had that, [...] My experience is that it is the colleagues' retention or insecurity, which is why not so much is substituted."
D	2		Bureaucracy in the prescription of controlled substances	"Basically, the prescriptions with controlled substances are more bureaucratic in every perspective and that may also be a reason why it is more difficult to substitute. […]."
D	3		Physicians and their expectations when describing controlled substances	"When it comes to controlled substances, less are prescribed in the form of active substances. Primary care physicians usually write the original on the prescription and rarely the generic. They also note down if they do not want the medicine substituted."
D	4		Pharmacy technicians' reluctance to address controlled substance generic substitution during a consultation	"Dealing with these strictly controlled substances is sometimes a bit difficult so that some people do not have enough confidence. I mean, we have pharmacy technicians and trainees; they are usually the people at the frontline, and if they don't have the confidence [...]. The thought of a separate law for controlled substances and that they should, therefore, be handled more strictly. [...]."
E	**Overarching content category**<br>Views indicating no challenges in substituting controlled substances			
E	**Nr.**	**Specific content subcategory**<br>Concerning community pharmacists' responses		**Example-Quote**
E	1		Statement about unproblematic generic substitution in controlled substances	"It is completely unproblematic; for example, we can substitute Targin with Oxycodone-Naloxone without any problems. Customers are open to that. There are individual cases, who want to stick with Targin, but in principle, it works well."
**Content group 3:**<br>**Main Question Topic:**<br>Support for the generic substitution of controlled substances				
F	**Overarching content category**<br>Support with the generic substitution in general			
F	**Nr.**	**Specific content subcategories**<br>Concerning community pharmacists' responses		**Example Quotes**
F	1		Generics in stock (guaranteed delivery)	"Here in this pharmacy, we ensure that we have the generics in stock whenever possible and not the originals, which often helps to ease the decision-making. Because people do not want to come back a second time or do not want to wait, because they are in pain now. Or they are frail or injured or in pain and want to go home into bed and not come back again. Then, we take what is available. So availability is the most important thing."
F	2		Standardized notes on the prescriptions	"A note, which says generics are desired would be good of course. Sometimes you even see prescriptions that explicitly state that a generic can be dispensed."

F	3		Information about co-marketing medicines	"The knowledge of the co-marketing list would also help if that were integrated into our pharmacy software. To identify what really is a co-marketing product would be extremely helpful. Then one can show that this generic medicine is one-to-one the same. I always say that the only difference is the packaging and the price. People understand that."
F	4		Better availability of generics	"I think it is important that generics have a place on the medicine market, that is undisputable. But then availability must be guaranteed and I know it is probably not always easy to guarantee this within the global market. However, we have an obligation, if we switch a patient to a generic, then we must also (be able to) supply them."
F	5		Similar specific properties of generics and original medicines	"It always helps the patient if the tablet looks the same or very similar; it makes it easier to understand that it is something similar; if they look relatively different, the skepticism is of course high. Especially for those people who know the original."
G	**Overarching content category**<br>Support with generic substitution in the treatment of nervous system disorders			
G	**Nr.**		**Specific content subcategories**<br>Concerning community pharmacists' responses	**Example Quotes**
G	1		Provided substitution information for pharmacy practice	"We have lists, for example, from the manufacturer […], which indicate sensitive products, like antiepileptics, where caution is needed, especially when they are used to treat epilepsy."
H	**Overarching content category**<br>Support with generic substitution of controlled substances			
H	**Nr.**		**Specific content subcategories**<br>Concerning community pharmacists' responses	**Example Quotes**
H	1		Official information about controlled substances	"It might help pharmacists if they were told that substitution of controlled substances is possible, even desirable to help reduce costs. That substitution is possible for controlled substances too."
H	2		Controlled substances quality, safety and equivalence based on study data	"[…] the safety through information and official guidelines that clearly state, that you can substitute without any problems."
H	3		Simplification of the additional administrative workload	"The question is, if we are allowed to write directly on the prescription for controlled substances or if a new one has to be sent? Yes, I would also need information on what I am officially allowed to do."
H	4		More time is required to get used to generic medicines of controlled substances	"Historically, however, there has clearly been a development. In the beginning, we simply did not substitute controlled substances. We needed time to gain trust."
H	5	**Nr.**	**Content sub-subcategories:**<br>Physicians and other service providers as important factors in the process	**Example Quote**
		1	More information from physicians regarding the comparability of original and generic medicines	"From the physicians' point of view, it would perhaps be useful if they also pointed out more to patients that generics are equivalent medicines, that this is where the information starts, people should already be informed. If the physician is already working towards this direction, it is also easier for us, I do not think that anything could be done additionally for list a opioids."
H	5	2	More cooperation between all service providers with regard to informing patients about possible generic medicines	"When the patient has questions about the physician's recommendation, we can simply explain why the physician prescribed the generic and why it makes sense. That way, the patient is more convinced that he is getting something good and tailored to his needs. […]."
